# The design of the run Clever randomized trial: running volume, −intensity and running-related injuries

**DOI:** 10.1186/s12891-016-1020-0

**Published:** 2016-04-23

**Authors:** Daniel Ramskov, Rasmus Oestergaard Nielsen, Henrik Sørensen, Erik Parner, Martin Lind, Sten Rasmussen

**Affiliations:** 1 Department of Public Health, Aarhus University, Aarhus, Denmark; 2 Orthopaedic Surgery Research Unit, Science and Innovation Center, Aalborg University Hospital, Aalborg, Denmark; 3 Department of Clinical Medicine, Aalborg University, Aalborg, Denmark; 4 Department of Public Health, Section for Biostatistics, Aarhus University, Aarhus, Denmark; 5 Department of Orthopaedics, Aarhus University Hospital, Aarhus, Denmark; 6 Department of Physiotherapy, University College Northern Denmark, Aalborg, Denmark

**Keywords:** Running, Musculoskeletal pain, Leg injuries, Athletic injuries, Recreational runners, Running schedule, Injury prevention, Running-related Injuries, Running volume, Running intensity

## Abstract

**Background:**

Injury incidence and prevalence in running populations have been investigated and documented in several studies. However, knowledge about injury etiology and prevention is needed. Training errors in running are modifiable risk factors and people engaged in recreational running need evidence-based running schedules to minimize the risk of injury. The existing literature on running volume and running intensity and the development of injuries show conflicting results. This may be related to previously applied study designs, methods used to quantify the performed running and the statistical analysis of the collected data. The aim of the Run Clever trial is to investigate if a focus on running intensity compared with a focus on running volume in a running schedule influences the overall injury risk differently.

**Methods/design:**

The Run Clever trial is a randomized trial with a 24-week follow-up. Healthy recreational runners between 18 and 65 years and with an average of 1–3 running sessions per week the past 6 months are included. Participants are randomized into two intervention groups: Running schedule-I and Schedule-V. Schedule-I emphasizes a progression in running intensity by increasing the weekly volume of running at a hard pace, while Schedule-V emphasizes a progression in running volume, by increasing the weekly overall volume. Data on the running performed is collected by GPS. Participants who sustain running-related injuries are diagnosed by a diagnostic team of physiotherapists using standardized diagnostic criteria. The members of the diagnostic team are blinded. The study design, procedures and informed consent were approved by the Ethics Committee Northern Denmark Region (N-20140069).

**Discussion:**

The Run Clever trial will provide insight into possible differences in injury risk between running schedules emphasizing either running intensity or running volume. The risk of sustaining volume- and intensity-related injuries will be compared in the two intervention groups using a competing risks approach. The trial will hopefully result in a better understanding of the relationship between the running performed and possible differences in running-related injury risk and the injuries developed.

**Trial registration:**

Clinical Trials NCT02349373 – January 23, 2015.

## Background

In modern society, running has become a popular way to exercise and the proportion of adults who participate in recreational running has been growing since the 1970s [1]. In surveys of participation in leisure time physical activity, running is reported to be one of the most popular forms of exercise [2–4] and a national report from the Danish Institute for Sports Studies, estimates that 31 % of the adult population in Denmark participate in recreational running [5]. Running is a cheap and easily accessible form of exercise, and the positive effects of running on health outcomes such as weight loss, cardio respiratory function and mortality are well known [6–8]. Unfortunately, running is also associated with the risk of sustaining a running-related injury. A recently published systematic review and meta-analysis by Videbæk et al. report, that the incidence of running-related injuries per 1000 h of running varies between 2.5 and 33.0 and estimates a weighted injury incidence among recreational runners of 7.7 (95 % CI 6.9 – 8.7) [9]. The knee, lower leg and ankle/foot are the most common sites of injury and a recent study revealed the median time to recovery from injury to be 72 days [10–13]. In addition, the proportion of injured runners receiving conservative treatment was 10.7 %, while 4.7 % underwent surgical treatment [10].

People engaged in recreational running or choosing running as a new and active lifestyle, should be offered evidence-based advice on running schedules with minimal injury risk. Running with a minimized risk of injury would aid the choice of an active lifestyle by decreasing discontinuation from running and, possibly, further reduce the proportion of people at risk of chronic diseases.

Training errors are recognized as a risk factor for running-related injuries and in order to develop running schedules with a minimized risk of injury, a better understanding of the different training variables’ influence on injury risk is needed [14, 15]. Trials investigating differences in injury risk in relation to the progression in running volume, the frequency and duration of running and participation in a preconditioning programme, have been conducted [16–18]. The GRONORUN 1 and 2 trials both mentions the application of an subjective measure of running intensity as a limitation in the trials [19, 20]. The study by Pollock et al.[18] used maximal oxygen uptake as a relative measure of running intensity, comparing different durations of running and different weekly running frequencies. However, the sample consisted of male prison inmates between 20 and 35 years of age, which makes it difficult to generalize the results to recreational or novice runners. A review by Nielsen et al. concluded that there are moderate evidence suggesting that volume is associated with injury risk, but that this association possibly is influenced by the weekly progression in volume and the maturation of the runners [15]. The existing literature on running intensity and the development of injuries show conflicting results, possibly related to the subjective measure used in previous studies [15]. A possible association between the focus of a running schedule and the risk of sustaining specific injuries has also been hypothesized. Specifically, patellofemoral pain, illiotibial band syndrome and patellar tendinopathy were hypothesized to be distance-related, while achilles tendinopathy, gastrocnemius injury and plantar fasciitis were hypothesized to be pace-related [21]. Findings from both experimental and cohort studies support this hypothesized association. In an experimental study by Petersen et al. [22], an increase in running speed resulted in an increase in the peak plantar flexion moment, which was significantly higher than the increase in the peak knee extensor moment (*p* < 0.05). In another experimental study by Petersen et al. [23], a decreased running speed resulted in a cumulative load at the knee joint which was significantly higher, compared with the cumulative knee joint load at higher running speeds [22, 23]. An observational study by Nielsen et al. [24], with a 1-year follow-up and 847 novice runners included, investigated the progression in volume and found that greater progression in running volume increased the risk of the above mentioned distance-related injuries, but not the overall risk of injury. The aim of the Run Clever trial is therefore to conduct a training schedule intervention trial, comparing a running schedule which focus on running intensity with a running schedule which focus on running volume, with the purpose of extending the above mentioned studies, by investigating the difference in risk between the two training variables and the risk of sustaining specific injuries associated with a specific training variable. The following hypotheses are tested (H).

1. Runners with a focus on running intensity have a 15 % increased risk of injury compared with runners with a focus on running volume.
2. A running schedule focusing on intensity, increase the risk of sustaining achilles tendinopathy, gastrocnemius injuries and plantar fasciitis compared with hypothesized distance-related injuries.
3. A running schedule focusing on running volume, increase the risk of sustaining patellofemoral pain syndrome, illiotibial band syndrome and patellar tendinopathy compared vwith hypothesized pace-related injuries.
4. A positive excess risk due to interaction exists between running intensity and running volume, and the effect is more pronounced for pace-related injuries with greater changes in speed than volume, while the effect is more pronounced for distance-related injuries with greater changes in volume than in speed.

Furthermore, complementary risk factor analysis on BMI, age, gender, previous injury, running experience and general activity level will be performed. Of interest is the effect modification of the mentioned risk factors on the interventions’ influence on injury risk. Explorative analyzes will be performed on data collected using the Oslo Sports Trauma Research Center Questionnaire [25]. The focus of the explorative analyzes is the reported physical complaints progressing to time loss injuries and the relationship with the running performed.

## Methods/design

**Fig. 1:**
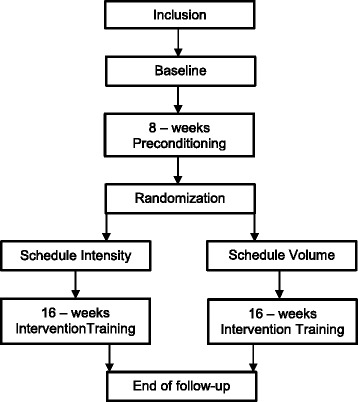
Flow-chart of outline of the RUN CLEVER trial

The Run Clever trial is a randomized trial with a 24 - week follow-up. The follow-up period is divided into an 8 - week *Preconditioning* period and a 16 - week *Intervention Training* period. The randomization is performed after the 8 - week preconditioning period (Fig. 1). Participants are randomly allocated into two intervention groups, following different running schedules: Schedule Intensity (Sch-I) and Schedule Volume (Sch-V). The main outcome is injury and the physiotherapists diagnosing injured participants (diagnostic team) are blinded to group allocation.

### Study population

The target population is recreational runners, defined as a person averaging between 1 and 3 weekly running sessions the past 6 months. Notice of the trial is distributed through social media, running magazines, recreational running clubs who are members of the Danish Athletic Federation (DAF), the Danish recreational sports association (DGI) and by handing out flyers at recreational running events. Persons interested, register at the trial homepage [26] and are asked to answer an online inclusion/exclusion questionnaire. Answers will be assessed by the investigators and eligible persons are included based on the inclusion and exclusion criteria.

### Inclusion and exclusion criteria

Healthy persons who own an IOS- or Android-based smart-phone, between 18 and 65 years, with an average of 1 – 3 weekly running sessions the past 6 months are eligible for inclusion. Persons will be excluded if they have had an injury in the lower extremity 6 months preceding the baseline, or if any of the following contraindications for vigorous physical activity are present: Former heart or chest surgery, symptoms of chest pain, dizziness or discomfort when physically active, pregnant or taking prescribed medication related to cardiovascular problems, in accordance with the American College of Sports Medicine (ACSM) [27].

### Data collection

At inclusion participants are provided access to a personal internet-based training diary and an IOS- or Android-based smart-phone application (Help2Run). The personal internet-based training diary is hosted by Amazon, and backed-up by a Help2Run server placed in Hornslet, Denmark. Continuously during follow-up, participants upload data on exposure (running), outcome (injury) and other co-variates (weight and general physical activity during daily life). Participants use the personal training diary to get an overview of completed training, answer administered questionnaires and report injuries. Through the back-end system of the internet based training diary, the research group access and download data on running performed, symptoms reported and follow-up measurements. All downloaded data is saved on the internal server hosted by Aarhus University. Access to the back-end system is granted to members of the research group, but only after permission has been granted by the principal investigator. The system complies with the proper privacy and confidentiality procedures of handling medical and personal information.

#### Data on exposure

Data on the running performed is collected using the smart-phone application. The smart-phone application is provided to the participant for free and synchronizes with the participants own smart-phone GPS, to collect the data on the running performed. A main feature of the smart-phone application is the live audio-feedback, provided to the participant regarding running pace. If a participant deviates from the scheduled running pace, by going too fast or too slow, the smart-phone application will provide an audio-feedback, until the participant is within the scheduled running pace.

#### Data on outcome

Outcome data is collected weekly using the Oslo Sports Trauma Research Center Questionnaire [25]. If full participation is not possible due to a perceived physical problem, the participant is contacted by telephone. If a participant reports an injury, an appointment for physical examination is made. Certified physiotherapists (the diagnostic team) perform the clinical examinations, using a previously employed standardized examination procedure [10]. Examinations are performed in the respective physiotherapeutic clinics, in which members of the diagnostic team are employed. After an examination, the physiotherapist reports data on diagnosis to the research group.

#### Baseline measurements

Participants complete the baseline measurements through the online training diary. The baseline measurements are divided into four parts which are administered consecutively.

1. is self-reported and covers personal information, demographics, medical history, information on running experience and the social security number. The self-reported height and weight will be used to calculate BMI (weight (kg)/height^2^ (m^2^)).
2. covers current symptoms of overuse injury, using a modified version of the Oslo Sports Trauma Research Center questionnaire (OSTRC) developed by Clarsen et al. [25], to establish a baseline measurement of overuse symptoms.
3. covers the baseline assessment of general physical activity during daily life. The Short Questionnaire to Assess Health-enhancing physical activity (SQUASH) is a tool, which assess habitual activity level during an ordinary week. Questions are pre-structured in four categories and information on days per week, average time and effort is collected. Time to complete the SQUASH questionnaire has been estimated to be 3 – 5 min [28].
4. and final part of the baseline measurement, is a field-based self-administered physical performance test (PPT). The participants are required to download the Help2Run smart-phone application, to complete the self-administered physical performance test. Through the smart-phone application the participants select 1 out of 3 possible running tests: 6 min running test, Coopers 12 min running test or 5 km running test. When the preferred test is completed, the test results are used to prescribe the intensity of running for each of the participants.

### Training variables

Four variables are used to design the running schedules for both intervention groups; frequency of running, progression in running, running volume and running intensity.

#### Running frequency

Running frequency is defined as the frequency at which participation in a series of stimuli per unit of time takes place [29]. Running frequency is quantified with each week as the time unit. The running schedule presents the running frequency, as the number of running session scheduled per week.

#### Running progression

Running progression is defined as the percentage increase in training load per unit of time [29]. The training load is quantified in kilometers with each week as the time unit. The running schedule presents the running progression as the percentage increase in kilometers between 2 weeks and as the percentage increase in kilometers between 2 and 4 week cycles.

#### Running volume

Running volume is defined as the total quantity of running [29] and is quantified in kilometers, combined with an indication of the time scale used. The running schedule presents the running volume as kilometers per session (km/session) and kilometers per week (km/week).

#### Running intensity

Running intensity is defined as the qualitative component of work performed in a given time [29]. The individual quantification of running intensity is divided into 3 intensities: easy pace ≈ 50–80%VO2max, moderate pace ≈ 81–87%VO2max and hard pace ≈ 88–100%VO2max [30]. The individual paces are presented as the minutes per kilometer (min/km) equal to the estimated VO2max, which are based on the results from the self-administered physical performance test, using the ${\overset{.}{V}}_{O_2}$ (VDOT) values presented by Daniels et al. [31, 32]. The running schedule presents the running intensity as kilometers per week per specific pace (km/week/pace).

### Intervention

**Fig. 2:**
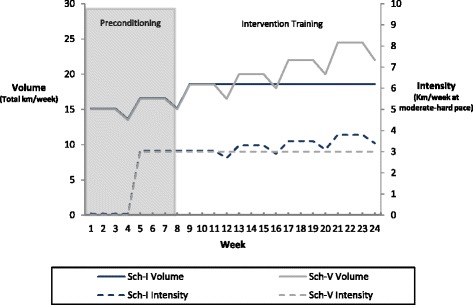
Development of the intervention during the entire follow-up. Volume (km/week) is the weekly total running volume. Intensity (km/week at moderate-hard pace) is the amount of kilometers at an intensity higher than 81 % VO2max. Schedule Intensity (blue) is focused on increasing the running intensity. Schedule Volume (grey) is focused on increasing the running volume

The training variables of interest are running intensity and running volume. Two running schedules have been designed, each with a specific focus on increasing either running intensity or running volume (Tables 1, 2 and 3). The training variables *running frequency* and *running progression* follow a similar fixed pattern in both training schedules throughout the 24-week follow-up. The 24 week running period consists of an 8 week preconditioning period and a 16 week intervention training period. The running frequency is 3 sessions per week for all weeks. Participants were advised to complete the weekly running sessions on the scheduled days (Tuesday – Thursday – Sunday), or preferably allow 1 day of recovery between running sessions. The overall progression in running follows a 4-week cycle. The 4-week cycle consist of a progression of 23 % in running (volume or intensity) the first week, a second and third adaptation week with 0 % progression and a fourth regression week with 10 % regression in running (Fig. 2).

**Table 1:** The content of the running schedule during the 8-week preconditioning period

Preconditioning period							
Training variables	Intensity	Volume	Frequency			Progression	
Week	km/week/pace	km/week	km session 1	km session 2	km session 3	prog/week	prog/cycle
1	15 easy	15	5	5	5	NA	NA
2	15 easy	15	5	5	5	0 %	
3	15 easy	15	5	5	5	0 %	
4	13,5 easy	13,5	3,5	5	5	−10 %	
5	13,5 easy/3 moderate	16,5	5	5	6,5 (3moderate)	23 %	10 %
6	13,5 easy/3 moderate	16,5	5	5	6,5 (3moderate)	0 %	
7	13,5 easy/3 moderate	16,5	5	5	6,5 (3moderate)	0 %	
8	12 easy/3 moderate	15	5	5 (3 moderate)	5 inc. PPT	−10 %	

The content is displayed in relation to the four training variables: intensity, volume, frequency and progression and for each of the 8 weeks. The kilometers to be completed in each running session are outlined in the collums of session 1–3. All kilometers are run at an intensity equal to easy pace, except if moderate or hard is specified (easy pace ≈ 50-80 %VO2max, moderate pace ≈ 81-87%VO2max and hard pace ≈ 88–100 %VO2max). The volume outlined in the parenthesis will then be completed in the specified pace. The progression is in total weekly volume. PPT is the abbreviation used for Physical Performance test

**Table 2:** The content of Schedule Intensity during the intervention training period

Intervention training period-schedule intensity							
Training variables	Intensity	Volume	Frequency			Progression	
week	km/week/pace	km/week	km session 1	km session 2	km session 3	prog/week	prog/cycle
1	15,5 easy/3 hard	18,5	5	7,5	6 (3 hard)	23 %	NA
2	15,5 easy/3 hard	18,5	5	6 (3 hard)	7,5	0 %	
3	15,5 easy/3 hard	18,5	5	7,5	6 (3 hard)	0 %	
4	13,8 easy/2,7 hard	18,5	5	6 (2,7 hard)	7,5	−10 %	
5	15,2 easy/3,3 hard	18,5	5	7,5	6 (3,3 hard)	23 %	9,50 %
6	15,2 easy/3,3 hard	18,5	5	6 (3,3 hard)	7,5	0 %	
7	15,2 easy/3,3 hard	18,5	5	7,5	6 (3,3 hard)	0 %	
8	15,6 easy/2,9 hard	18,5	5	6 (2,9 hard)	7,5 inc. PPT	−10 %	
9	15 easy/3,5 hard	18,5	5	7,5	6 (3,5 hard)	23 %	6 %
10	15 easy/3,5 hard	18,5	5	6 (3,5 hard)	7,5	0 %	
11	15 easy/3,5 hard	18,5	5	7,5	6 (3,5 hard)	0 %	
12	15,4 easy/3,1 hard	18,5	5	6 (3,1 hard)	7,5	−10 %	
13	14,7 easy/3,8 hard	18,5	5	7,5	6 (3,8 hard)	23 %	9 %
14	14,7 easy/3,8 hard	18,5	5	6 (3,8 hard)	7,5	0 %	
15	14,7 easy/3,8 hard	18,5	5	7,5	6 (3,8 hard)	0 %	
16	15,1 easy/3,4 hard	18,5	5	6 (3,4 hard)	7,5 inc. PPT	−10 %	

The content is displayed in relation to the four training variables: intensity, volume, frequency and progression and for each of the 16 weeks. The kilometers to be completed in each running session are outlined in the collums of session 1 – 3. All kilometers are run at an intensity equal to easy pace, except if moderate or hard is specified (easy pace ≈ 50–80%VO2max, moderate pace ≈ 81–87%VO2max and hard pace ≈ 88–100%VO2max). The volume outlined in the parenthesis will then be completed in the specified pace. The progression is in total weekly volume at a hard pace. PPT is the abbreviation used for Physical Performance test

**Table 3:** The content of Schedule Volume during the intervention training period

Intervention training period-schedule volume							
Training variables	Intensity	Volume	Frequency			Progression	
week	km/week/pace	km/week	km session 1	km session 2	km session 3	prog/week	prog/cycle
1	15,5 easy/3 moderate	18,5	5	5	8,5 (3 moderate)	23 %	11,50 %
2	15,5 easy/3 moderate	18,5	5	8,5 (3 moderate)	5	0 %	
3	15,5 easy/3 moderate	18,5	5	5	8,5 (3 moderate)	0 %	
4	13,5 easy/3 moderate	16,5	5	6,5 (3 moderate)	5	−10 %	
5	17 easy/3 moderate	20	5	6	9 (3 moderate)	23 %	8,50 %
6	17 easy/3 moderate	20	5	9 (3 moderate)	6	0 %	
7	17 easy/3 moderate	20	5	6	9 (3 moderate)	0 %	
8	15 easy/3 moderate	18	5	83 (3 moderate)	5 inc. PPT	−10 %	
9	19 easy/3 moderate	22	5	7	10 (3 moderate)	23 %	10 %
10	19 easy/3 moderate	22	5	10 (3 moderate)	7	0 %	
11	19 easy/3 moderate	22	5	7	10 (3 moderate)	0 %	
12	17 easy/3 moderate	20	5	9 (3 moderate)	6	−10 %	
13	21,5 easy/3 moderate	24,5	5	8	11,5 (3 moderate)	23 %	11 %
14	21,5 easy/3 moderate	24,5	5	11,5 (3 moderate)	8	0 %	
15	21,5 easy/3 moderate	24,5	5	8	11,5 (3 moderate)	0 %	
16	19 easy/3 moderate	22	5	10 (3 moderate)	7 inc. PPT	−10 %	

The content is displayed in relation to the four training variables: intensity, volume, frequency and progression and for each of the 16 weeks. The kilometers to be completed in each running session are outlined in the collums of session 1 – 3. All kilometers are run at an intensity equal to easy pace, except if moderate or hard is specified (easy pace ≈ 50–80%VO2max, moderate pace ≈ 81–87%VO2max and hard pace ≈ 88–100%VO2max). The volume outlined in the parenthesis will then be completed in the specified pace. The progression is in total weekly volume. PPTis the abbreviation used for Physical Performance test

#### Preconditioning period

All included participants follow the same running schedule during the 8 week preconditioning period. The running frequency is 3 running sessions per week, the progression in running volume follows the outlined structure of the 4-week cycle and all running will be performed at an intensity equal to an easy or moderate pace.

#### Intervention training period

*Sch*-*I* (Intensity training group) is exposed to increased running volume at a hard pace (defined as an intensity equal to or above a VO2max of 88 %). The total running volume per week is fixed, and only kilometers at a hard running pace follow the progression in running. The running volume at a hard pace will increase in each 4 week progression cycle (Fig. 2). The remaining distance will be performed at an easy pace (defined as an intensity below a VO2max of 80 %).

*Sch*-*V* (Volume training group) is focused on increasing the total running volume per week. All scheduled running will be performed at either an easy or moderate pace (defined as an intensity equal to or below a VO2max of 88 %). Only the total running volume per week follows the progression in running (Fig. 2).

The intervention in both groups is presented in Fig. 2 and Tables 1, 2 and 3.

### Follow-up

Follow-up measurements collected are weight, OSTRC injury severity score [33], SQUASH questionnaire [28] and self-administered performance tests.

**Fig. 3:**
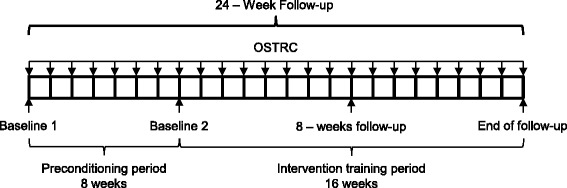
Follow-up measurements during the 24 weeks. The OSTRC questionnaire is administered every Sunday. Baseline 1, is the measurements collected at inclusion. Baseline 2, 8 weeks follow-up and End of follow-up is the follow-up measurements

The OSTRC questionnaire is administered every Sunday on a weekly basis. If a participant has not answered the OSTRC questionnaire the following Monday, an automated reminder e-mail is forwarded, requesting an answer. At the end of the first 8 weeks of follow-up, the preconditioning period ends, and follow-up measurements are collected. These measurements are also used as baseline measurements for the following 16 weeks intervention training period (baseline 2). During the intervention training period, follow-up measurements are collected 2 times: after 8 weeks and at end of follow-up (Fig. 3).

### Randomization

The randomization procedure is applied through a random sequence allocation in the back-end system of the internet-based training diary. The algorithm applied ensures equal group size. Participants are allocated to schedule *Sch*-*I* or *Sch*-*V* at inclusion, using concealed even or odd numbers generated by the back-end system, which is only accessible by the investigators. The group allocation is also concealed to the investigator, until after inclusion of a participant, as the number allocation is based on, is generated after a participant is included. The diagnostic team assessing injuries (outcome) is blinded to group allocation when collecting data on outcome.

### Outcome

The primary outcome is injury defined as a Running-Related Injury (RRI): *An injury sustained on muscles, joints, tendons and/or bones during or after running and attributed to running. The injury must have caused a training reduction (reduced distance, intensity, frequency etc.) for at least 7 days* [19], [34].

The secondary outcome is symptoms of overuse injury defined as: *A physical problem perceived as pain, tenderness, stiffness, aching, looseness, locking or instability in any part of the body* [25]. The information is collected using a modified version of the OSTRC questionnaire [25]. The modification of the questionnaire consists of an addition to the possible answers in the fourth and last question *“Cannot participate due to pain”*. The included anatomical areas are: foot, ankle, front of lower leg, calf, knee, thigh, hamstrings, groin, glutes, hip and lower back. An approach similar to the one used by Clarsen et al. [33], is applied to ease the response load on the participants.

All participants who report an RRI are referred to injury diagnosis, performed by a physiotherapist. Injury diagnosis is carried out using a standardized examination protocol and standardized diagnostic criteria [10]. The OSTRC questionnaire will continue to be administered to participants who are diagnosed with an injury, in order to explore the severity of injuries.

### Power

The power calculation was based on a superiority calculation. Based on comparisons between the current and previous studies, considering differences between populations and interventions, and including experiences from clinical practice, an injury incidence of 20 % is expected in runners focusing on volume and an injury incidence of 35 % is expected in runners focusing on intensity [18, 19]. To be able to show a minimum difference in injury risk between groups of 5 %, a sample size of 620 participants is required to reach a power of 80 %. An accommodation to a potential loss to follow-up is necessary to include in determining the number of participants needed. In prospective studies with a self-structured running regime and a follow-up ≥ 6 months, the loss to follow-up have been reported to be approximately 22–30 % [34, 35]. Prospective studies with a structured running regime, and follow-up periods of 13 weeks, reported the loss to follow-up to be approximately 8 and a 60 % completion of the scheduled running [12, 19]. Taking into consideration the duration of the 24-week follow-up period in the present study, and the motivational factor possibly associated with a structured running regime, a potential loss to follow-up of 15 % is hypothesized, which leads to a required sample size of 713 participants.

### Statistical analysis

All data will be analyzed using an instrumental variable approach in the primary analysis [36]. The instrumental variable is the randomization. Secondary analyzes are intention-to-treat and per-protocol. Time to first injury is analyzed using the following primary time scale: calender time in days and weeks. Data will be analyzed at the following time-points: 2 weeks, 4 weeks, 8 weeks and 16 weeks after randomization. Secondary time scales are cumulative running volume in kilometres and cumulative running volume in minutes. Time of follow-up begins when the randomized interventions start. The unit of analysis is each participant. Participants will be right-censored in case of pregnancy, disease, lack of motivation, non-running-related injury causing a permanent stop of running, unwillingness to attend clinical examination in case of injury, if more than 10 % of all training sessions were uploaded manually (they will be censored at the time 10 % of uploads are manual) or at end of follow-up, whichever comes first.

The injury proportion as a function of the follow-up time scales mentioned above will be estimated using the Kaplan-Meier curve. The cumulative injury risk difference between groups is analyzed, performing a generalized linear regression, using the pseudo values method [37]. Competing risk analysis will be performed separately for both groups, to investigate difference in risk of hypothesized injuries including more than one endpoint [38]. The Aalen Johansen estimator will be applied in the competing risks analysis. Endpoints included in the competing risk analysis are non-running-related injury causing a permanent stop of running and the 20 injuries presented by Taunton et al. [13] In the intensity group (Sch-I) the risk of achilles tendinopathy, gastrocnemius injuries, and plantar fasciitis are analyzed in a competing risk model, treating the remaining 17 injuries as competing events. In the volume group (Sch-V) the risk of patellofemoral pain syndrome, iliotibial band syndrome, and patellar tendinopathy are analyzed in a competing risk model, treating the remaining 17 injuries as competing events [21]. Differences are considered statistically significant at *p* < .05, and estimates are presented with 95 % confidence intervals. All analyzes are performed using STATA/SE version 13 (or more if applicable).

### Complementary risk factor analysis

To study if the effect of the intervention on the risk of RRI were modified by running experience, BMI, gender, previous injury, age and general activity level, a stratified analysis in accordance with the recommendations by Knol, *MJ* and Vanderweele, *TJ* [39] will be performed. All predictor variables will be included in the stratified analysis, provided that the recommendation of at least 10 injuries per predictor variable is fulfilled [40].

## Discussion

Run Clever is the first randomized trial to investigate how a running schedule focused on running intensity compared with a running schedule focused on running volume influences the overall risk of injury and the type of injuries sustained.

The population of interest is rapidly growing and should be prioritized, as it possibly is the largest group of recreational active adults worldwide [1, 41]. Recreational runners as a group are characterized by a large heterogeneity, especially concerning running experience and training habits. Consensus regarding the definition of a recreational runner is not established and the term is used inconsistently in relation to a wide variety of running populations. In three studies all describing the included runners as recreational, running experience in years ranged from 5.0 to 8.6 and the weekly volume in kilometers ranged from 28 to 38. However, the weekly running frequency only ranged from 3.0 to 3.8 [35, 42, 43]. The present trial defines the population as recreational runners, using the inclusion criteria of an average weekly frequency between 1 and 3 running sessions the past 6 months. The choice of weekly frequency was primarily based on a descriptive study by the Danish Institute for Sports Studies, which showed that the largest proportion of recreational runners in Denmark averaged 1 – 3 weekly running sessions [44].

The ideal percentage of weekly progression is unclear and a trial by Buist et al. comparing a weekly progression of 10.5 and 23.7 % found no difference in risk of RRI. However, a weekly progression of 30 % has been found to increase the risk of specific RRI [19, 24]. In an attempt to allow for adaptation to the progression in running, the research team developed the 4-week cycle, based on a theoretical step loading approach [29].

The follow-up Run Clever applies, aims at increasing the homogeneity of the included participants, in relation to the running performed most recently, prior to allocation to the intervention. Other running-related factors, such as running experience and previous running-related injury, are non-modifiable parts of the heterogeneity characterizing recreational runners, and can only be sought controlled for through the baseline data collection and complementary risk factor analysis.

The superiority power calculation based on a minimum risk difference between groups of 5 %, and taking a hypothesized loss to follow-up of 15 % into account, revealed a required sample size of 713 participants to reach a power of 80 %. A predetermined inclusion period of 6 months has been determined. The challenge of including 713 participants over a 6 month period is acknowledged. However, the estimated 31 % of the adult population in Denmark who participates in recreational running, provides an approximate background population of 904.685 persons, from which recruitment of participants can take place (Based on numbers from Statistics Denmark) [45]. Information regarding the possibility of participation in the trial is announced through the two largest Danish recreational running organizations. Possible participants can easily access and fill out the inclusion/exclusion questionnaire and indicate their interest in participation. The baseline information is collected through the online training diary, making data collection less time consuming for both participants and investigators. This gives us reason to assume that such a large scale inclusion is possible. However, a sample size of 357 participants, based on a minimum risk difference between groups of 1 %, with a power of 80 % and taking the hypothesized loss to follow-up of 15 % into account, is the minimal acceptable number of included participants and also the minimal acceptable clinical relevant difference. At the end of the 6 month inclusion period, no additional inclusions will be made.

The use of GPS to collect data on running exposure is not new and the applicability of this method has been investigated both in runners and in field-based sports [46–50]. In the current trial, GPS is used to quantify volume (kilometers) and intensity (minutes per kilometer). Nielsen et al. [47] investigated the feasibility of using GPS to quantify training volume in kilometers. The study concluded that the use of GPS caused no clinically relevant measurement errors. Townshend et al. [46] investigated the accuracy of GPS in the measurement of running speed. The study concluded that the use of GPS can provide accurate data on running speed. Further, unpublished data found a Root Mean Square (RMS) of the average pace in minutes per kilometer < 5 s per kilometer, when collected over approx. 300 m. If the distance was increased to 1000 m, the RMS of the average pace in minutes per kilometer was 3 – 5 s per kilometer [51]. Based on this information, it seems feasible to use GPS to quantify running exposure data on running volume (kilometers) and running intensity (minutes per kilometers). Running intensity will, however, not be averaged using distances < 500 m in the current trial.

A modification of the fourth and last question in the OSTRC questionnaire [25], used to collect data on the secondary outcome is applied. The original severity score ranging from 0 to 100 is not changed. In order to accomplish this, the added fifth answering possibility is given the same response value as the fourth answering possibility. The modified version was employed based on experiences from an ongoing study in our lab on handball injuries, providing previously injured respondents with a possibility of reporting “no participation due to pain”.

### Ethics and consent to participate

The study design, procedures and informed consent were approved by the Ethics Committee Northern Denmark Region (N-20140069) and the Danish Data Protection Agency. All included participants provided informed verbal and written consent.

### Consent to publish

Not applicable.

### Availability of data and materials

Dataset (STATA. dta file or excel file), statistical code (STATA. do file) and codebook (.pdf) of published findings can be requested by contacting the corresponding author. Participants did not provide informed consent to data sharing. All data will therefore be anonymized and personal information will be deleted before data sharing.

## Abbreviations

ACSM: The American College of Sports Medicine
DAF: the danish athletic federation
DGI: the danish recreational sports association
OSTRC: The Oslo Sports Trauma Research Center
PPT: physical performance test
RRI: running-related injury
Sch-I: running schedule which focuses on running intensity
Sch-V: running schedule which focuses on running volume
SQUASH: the short questionnaire to assess health-enhancing physical activity
